# Application of the process-based teaching based on SPARK case database in the practice teaching of radiology in the musculoskeletal system for undergraduate medical students

**DOI:** 10.1186/s12909-024-05672-z

**Published:** 2024-06-22

**Authors:** Yangsheng Li, Zhijiang Han, Qianqian Xia, Chengcheng Gao, Chunjie Wang, Xiangwen Zhu, Zhongxiang Ding, Jiying Zhu

**Affiliations:** Department of Radiology, Affiliated Hangzhou First People’s Hospital, Westlake University School of Medicine, No.261, Huansha Road, Hangzhou, Zhejiang 310006 China

**Keywords:** Medical education, The process-based teaching, SPARK case database, Teaching of radiology, The musculoskeletal system

## Abstract

**Background:**

Process-based teaching is a new education model. SPARK case database is a free medical imaging case database. This manuscript aimed to explore the application of the process-based teaching based on SPARK case database in the practice teaching of radiology in the musculoskeletal system.

**Methods:**

117 third year medical students were included. They were divided into Group A, B, C and D according to the curriculum arrangement. Group A and B attended the experimental class at the same time, A was the experimental group, B was the control group. Group C and D attended experimental classes at the same time, C was the experimental group, D was the control group. The experimental group used SPARK case database, while the control group used traditional teaching model for learning. The four groups of students were respectively tested after the theoretical class, before the experimental class, after the experimental class, and one week after the experimental class to compare the results. Finally, all students used SPARK case database to study, and were tested one month after the experimental class to compare their differences.

**Results:**

The scores after the theoretical class of Group A and B were (100.0 ± 25.4), (101.0 ± 23.8)(*t*=-0.160, *P* > 0.05), Group C and D were (94.7 ± 23.7), (92.1 ± 18.6)(*t* = 0.467, *P* > 0.05). The scores of Group A and B before and after the experimental class and one week after the experimental class were respectively (84.1 ± 17.4), (72.1 ± 21.3)(*t* = 2.363, *P* < 0.05), (107.6 ± 14.3), (102.1 ± 18.0)(*t* = 1.292, *P* > 0.05), (89.7 ± 24.3), (66.6 ± 23.2)(*t* = 3.706, *P* < 0.05). The scores of Group C and D were (94.0 ± 17.3), (72.8 ± 25.5)(*t* = 3.755, *P* < 0.05), (107.3 ± 20.3), (93.1 ± 20.9)(*t* = 2.652, *P* < 0.05), (100.3 ± 19.7), (77.2 ± 24.0)(*t* = 4.039, *P* < 0.05). The scores of Group A and B for one month after the experimental class were (86.6 ± 28.8), (84.5 ± 24.0)(*t* = 0.297, *P* > 0.05), and Group C and D were (95.7 ± 20.3), (91.7 ± 23.0)(*t* = 0.699, *P* > 0.05).

**Conclusions:**

The process-based teaching based on SPARK case database could improve the radiology practice ability of the musculoskeletal system of students.

## Introduction

At present, traditional teaching method occupies a dominant position in Chinese medical education. The form and content of this one-way learning method are mechanical and obsolete, which is not conducive to cultivating students’ clinical comprehensive ability [1]. At the same time, the traditional teaching method pays more attention to the result than the process, takes the final examination results as the most important indicator to measure the teaching effect, and pays no attention to the learning process, which not only reduces the enthusiasm and participation of students, but also easily leads to the situation of cramming at the end of the semester. Radiology is one of the compulsory courses for undergraduate medical students. This subject is highly specialized, so it is difficult to be fully digested in the limited classroom time. Meanwhile, some students lack learning motivation and self-control. The above reasons lead to a great discount in the quality of undergraduate radiology teaching. Obviously, traditional teaching method can no longer meet the needs of modern society. Whitehead, an outstanding educator in the 20th century, put forward the philosophy of process and believed that we should attach importance to the process of education [2]. In China, compared with the previous “target teaching” and “final performance assessment”, teachers are increasingly required to focus on the process-based teaching. This is a new type of education and training model with stages and levels. Teachers are required to guide and supervise every link of students’ learning, and set up stage assessment for each node, so as to dynamically evaluate the learning effect of students, avoid major omissions and deviations, and ensure teaching quality. Obviously, textbooks and traditional classroom cannot achieve this goal, so how to find a suitable way to achieve process-based teaching is an urgent problem for us to solve. The outbreak of COVID-19 has made people turn their attention to online teaching [3, 4], which has good teaching effects, playing an irreplaceable role during epidemic prevention and control [5–8]. At the same time, the emergence of online teaching enables teachers to monitor and evaluate students’ learning process in real time. It promotes the transformation of teaching from purposive to procedural.

In medical imaging, learning a complete system is also the embodiment of process-based teaching. The transition from superficial to profound, theoretical knowledge to clinical practice requires the speciality of sub-speciality(S), problem-based learning(P), assessment(A), report (R) and reading skill(K), which we summarized as “SPARK“ [9]. Meanwhile, the corresponding software platform has been developed, and the case database of public welfare medical imaging has been established. This platform is open to everyone for free. It has been applied to the acute abdomen radiology teaching for the first time, and has achieved satisfactory results [9]. The proposal and establishment of the case database provide more possibilities for the development and inheritance of radiology.

The musculoskeletal system is an important part of radiology. In this study, we used SPARK case database to apply the process-based teaching to the teaching of musculoskeletal system of students, and compared it with the traditional teaching model, so as to evaluate the teaching application effect of both.

## Materials and methods

### Participants

This experiment included 117 third year students majoring in Clinical Medicine from the Fourth Clinical Medical College of Zhejiang University of Traditional Chinese Medicine as the research subjects. All students were arranged to participate in medical imaging courses in the first half of 2022, and each student voluntarily participated in the experiment. This experiment was a prospective controlled trial, but not a randomized trial. According to the division of administrative classes in the school, we divided clinical major classes 1, 2, 3, and 4 into groups A, B, C, and D. The school arranged teaching on an administrative class basis and it arranged for Group A and Group B to conduct experimental classes simultaneously, selecting Group A as the experimental group and Group B as the control group. Group C and Group D were given experimental classes at another time simultaneously. Group C was selected as the experimental group and Group D as the control group. Written informed consent was obtained from all participating students.

### Inclusion and exclusion criteria

Inclusion criteria: ①All students in each group participated in the teaching of the theoretical and experimental classes and completed the tests in each stage; ②The completion rate of SPARK musculoskeletal question set of students in the experimental group exceeded 90%. Exclusion criteria: ①Students were absent from any or both of the theoretical or experimental classes, or failed to complete any stage of the test; ②Students quit the experiment voluntarily; ③The completion rate of SPARK musculoskeletal question set of students in the experimental group was no more than 90%.

### Study design

According to the school curriculum arrangement, the four groups of students first studied the theoretical class of musculoskeletal system at the same time. The content of the course was mainly taught by the teacher on musculoskeletal system related theoretical knowledge, totaling 4 class hours. After the theoretical class, each group would be assigned corresponding learning tasks to study separately. After 3 days of study, Group A and B had the experimental class. Group C and D were carried out 10 days later. According to the syllabus, the course included knowledge points related to bone and joint injury, chronic osteoarthropathy and common bone tumors. Among them, chronic osteoarthritis mainly included degenerative changes, ankylosing spondylitis, bone tuberculosis, osteomyelitis and rheumatoid arthritis. Common bone tumors mainly included osteosarcoma, osteochondroma, giant cell tumor of bone, bone cyst, and bone metastasis.

### Teaching methods of the experimental group

**Fig. 1:**
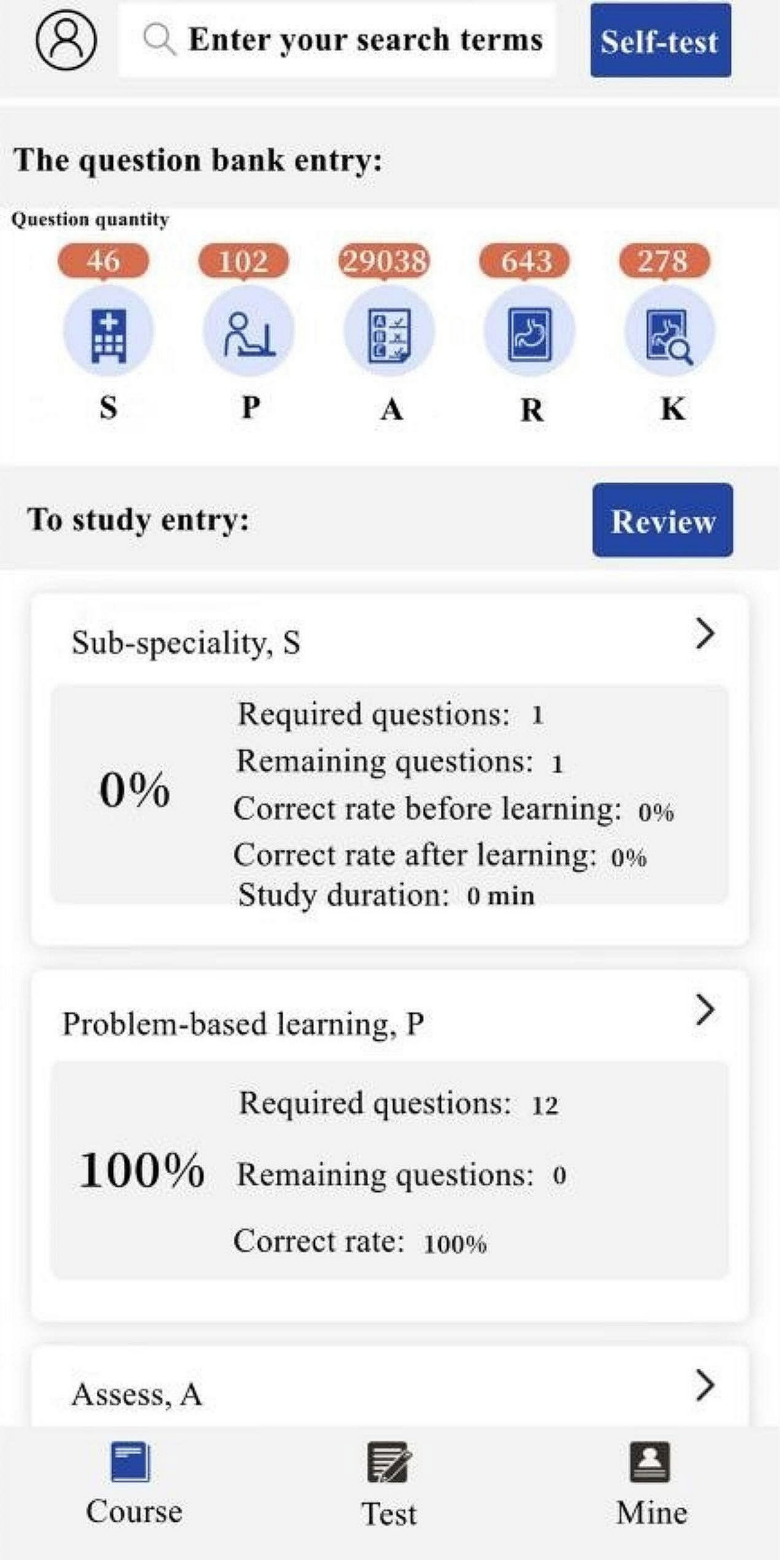
SPARK case database Home Page part 1

**Fig. 2:**
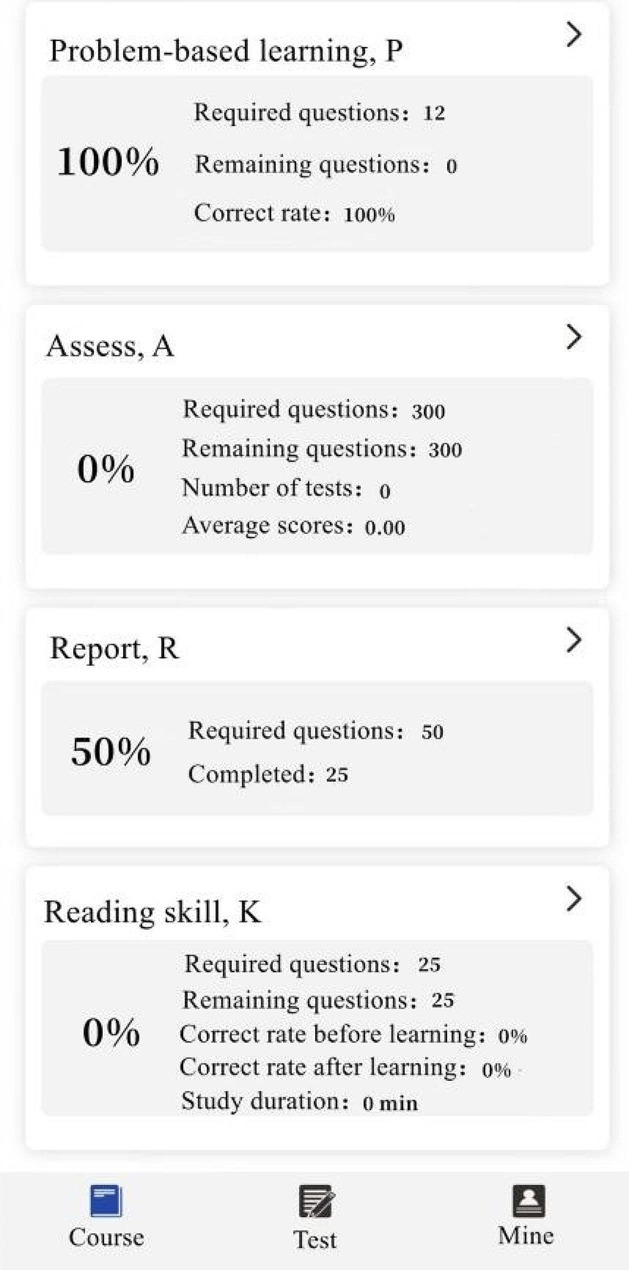
SPARK case database Home Page part 2

**Fig. 3:**
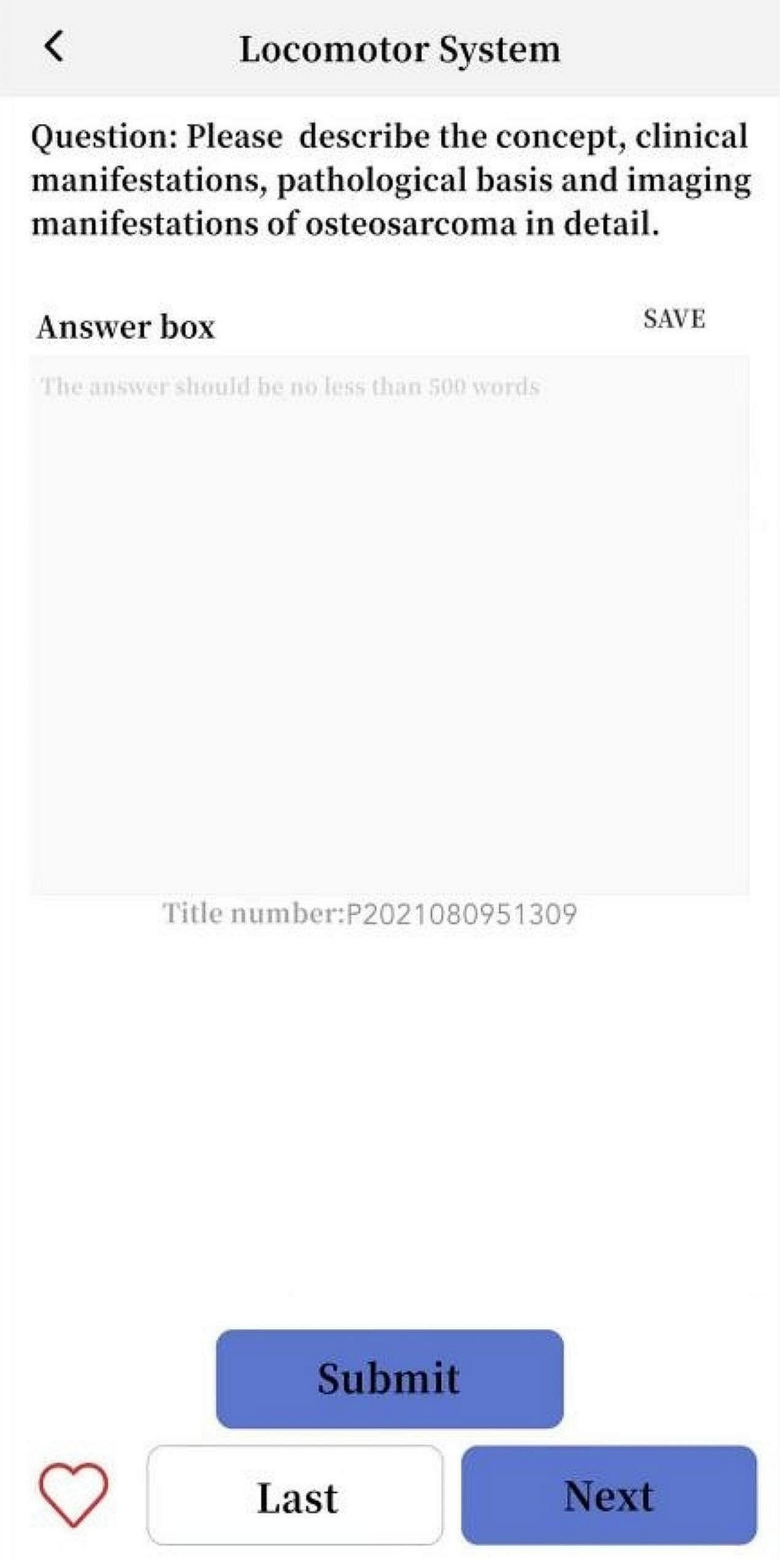
A schematic diagram of Part P in the SPARK case database

**Fig. 4:**
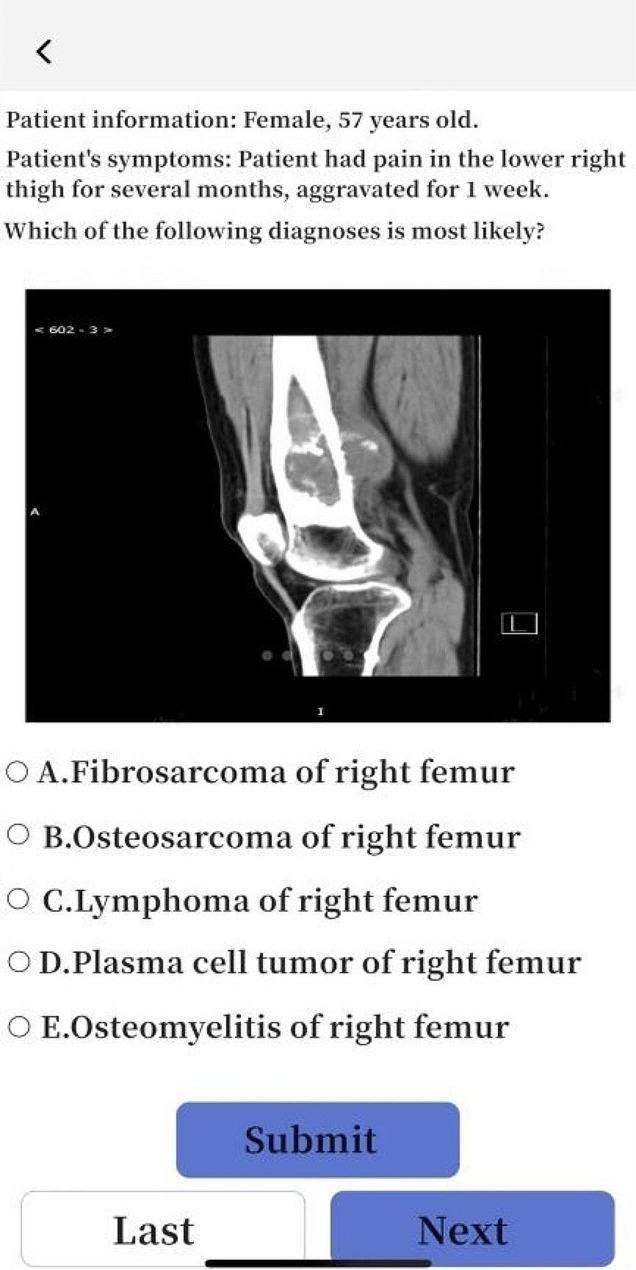
A schematic diagram of Part A in the SPARK case database

**Fig. 5:**
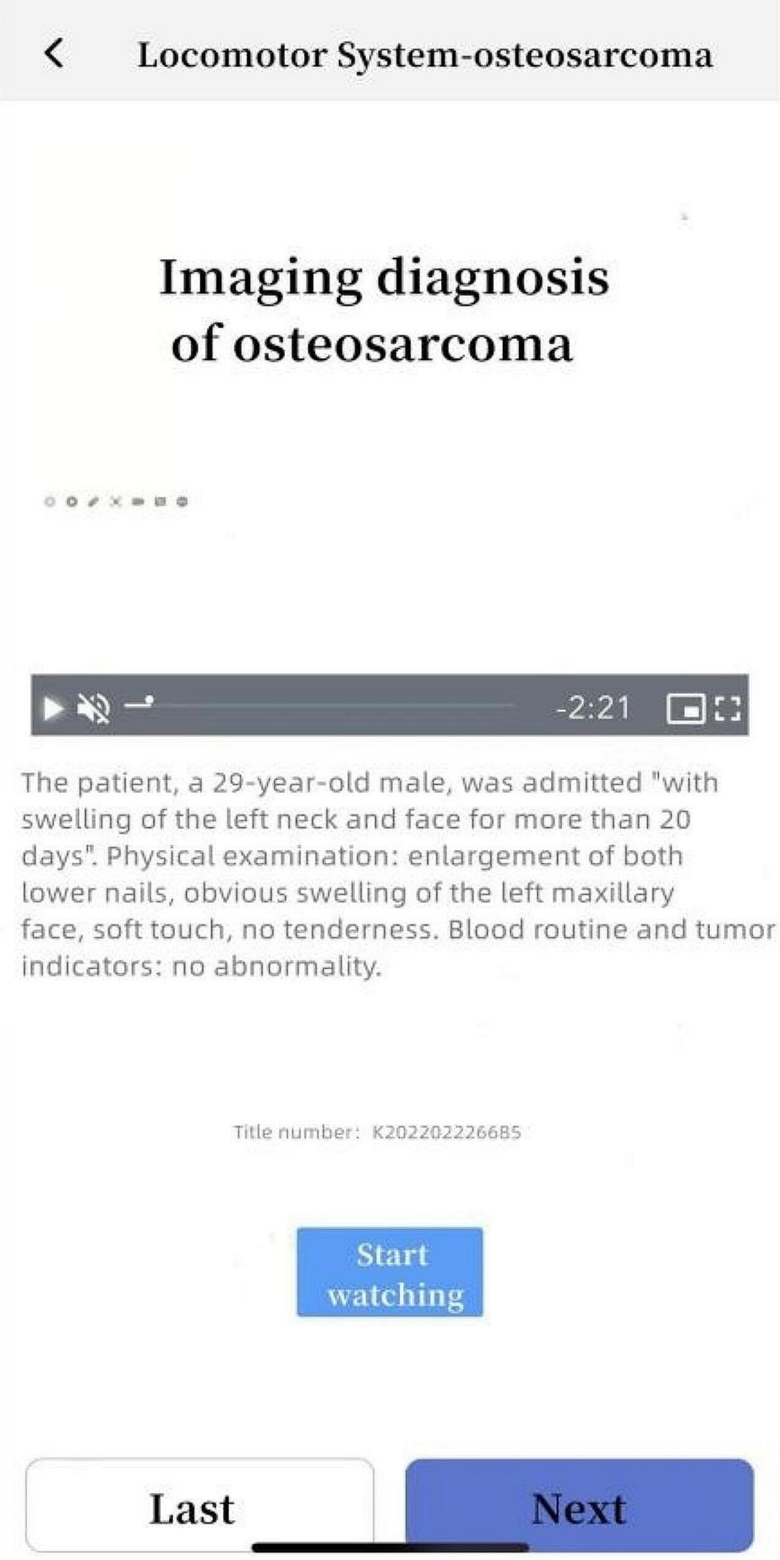
A schematic diagram of Part K in the SPARK case database

Students in Group A and Group C used SPARK case database for after-class learning. After the theoretical class, the teacher assigned the same set of musculoskeletal system questions to each student through the software backstage. The learning time of Group A was 3 days, and that of Group C was 10 days. The question set consisted of the following five modules: ①Sub-speciality(S): The students watched the online musculoskeletal lecture provided by senior imaging doctors for about 50 min, including the knowledge points to be mastered for musculoskeletal system; ②Problem-based learning(P): One short answer question was set for each disease of the musculoskeletal system. For example, please describe the concept, clinical manifestations, pathological basis and imaging manifestations of osteosarcoma in detail. Students needed to consult books and fill in the answer box with the correct answer through voice to text conversion or manual text input. Copying and pasting was invalid (To prevent students from searching for answers online and directly copying and pasting them into the answer box). Only when the accuracy of answers detected by the background exceeded 80%, could it be completed and submitted; ③Assessment(A): 300 interpretation questions of the best imaging results of the musculoskeletal system were set, and students needed to select the best choice according to the pictures(most of the questions had been confirmed by pathology, except for fractures and other diseases that did not need to be confirmed by pathology); ④Report(R): This link was set with 50 musculoskeletal system report writing questions. The key words in the report were set as drop-down box options. Students could submit the answers only after selecting the correct one; ⑤Reading skill(K): 2–3 short videos were set for each musculoskeletal system diseases, with the duration of about 2–3 min. Each video was based on the real clinical cases, and the general information, present history, laboratory examination, physical examination and imaging examination of the patients were comprehensively analyzed. For some contents of SPARK case database, see Figs. 1, 2, 3, 4, 5.

After 3 days and 10 days of study, each group had the experimental class. After the end of the experimental class, the above two experimental groups were still assigned the SPARK question set. The modules and quantity were the same as before. The contents were adjusted according to the results of the two tests before and after the experimental class, and the proportion of unmastered diseases would be appropriately increased. Group A and Group C would continue to study for one week. The teacher observed the students’ learning progress in the background of the software during the two learning periods and urged them to complete as many learning tasks as possible.

### Teaching methods of the control group

The students in Group B and Group D studied by traditional teaching methods after class. The teacher assigned learning tasks in the Wechat communication group after the theoretical and experimental classes, and the students studied by reading the textbook part of musculoskeletal system and teaching courseware(WeChat is a communication software mainly for daily conversations, which is widely used in China). The time and learning duration of the two tasks assigned by Group B were the same as those of Group A, while Group D was the same as Group C. Teachers urged students to study through Wechat communication groups during the two study periods.

### Teaching methods of the experimental class

Before the experimental class, the teacher arranged the students of Group A and B, Group C and D to take a test. Then the teacher counted the number of correct answers in each part of the examination paper, adjusted the class content according to the results, analyzed and explained the deficiencies of the students, and made up for loopholes in their knowledge points. After the experimental class, the same examination paper was used for the test again, and the real-time listening effect of the students was evaluated according to the results on both sides. At the same time, questionnaires were issued to calculate the average daily learning time of students at this stage.

**Fig. 6:**
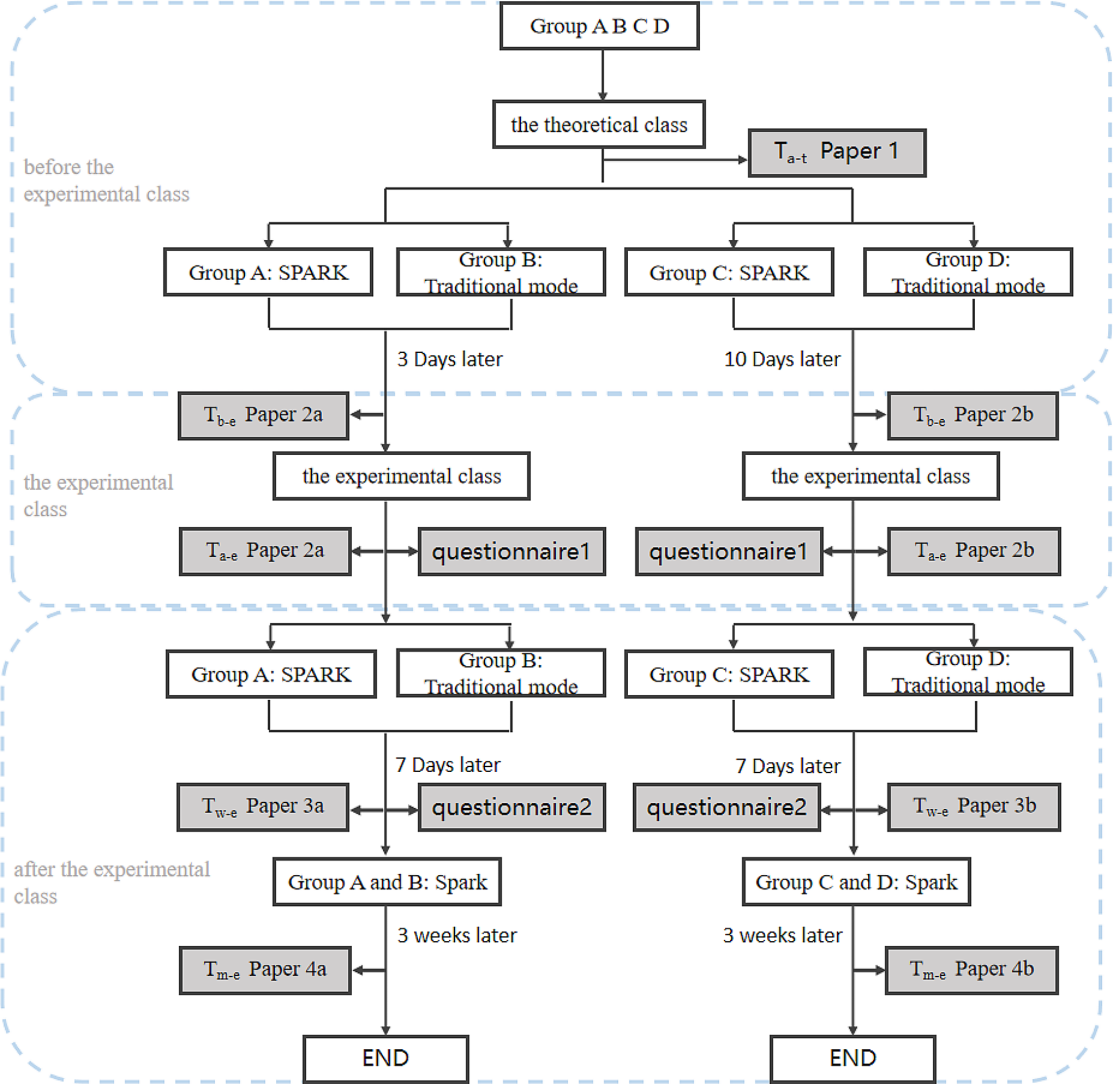
Experimental design flow chart

**Fig. 7:**
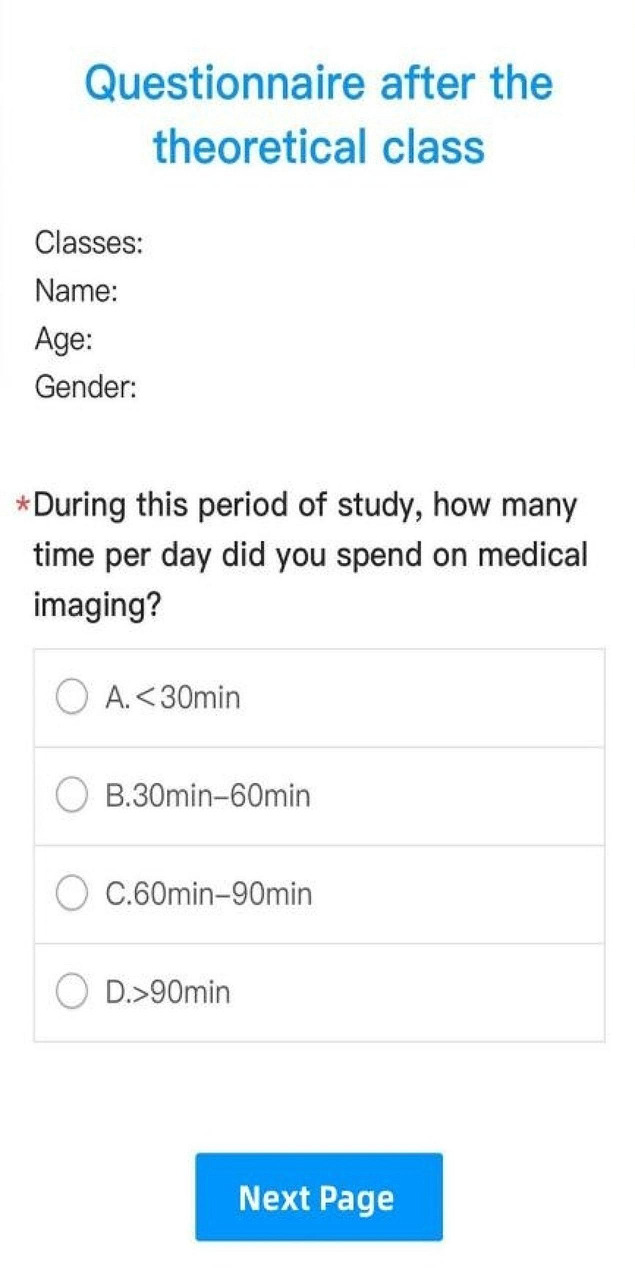
A schematic diagram of the first questionnaire

After the experimental class, the students continued to study using their own learning model. After one week, we arranged another test to evaluate the learning effect of them in this week and gave out questionnaires. Finally, in order to ensure the fairness of teaching, all students were assigned SPARK musculoskeletal system question sets after the above test, and the final test was conducted one month after the experimental class in each group to assess whether performance gaps remain. The experimental flow chart was shown in Fig. 6. The questionnaire diagram was shown in Fig. 7.

### Data evaluation and statistical analysis

The students in Group A and B, C and D were tested after the theoretical class(T_a−t_), before the experimental class(T_b−e_), after the experimental class(T_a−e_), one week after the experimental class(T_w−e_) and one month after the experimental class(T_m−e_) respectively. The results of each test were recorded. Meanwhile, the number of correct answers in each part of the two tests before and after experimental class were counted. In the test after the theoretical class, the test papers of the 4 groups were the same. And papers of the other testing stages were different, except the test papers after the experimental class. Because it was the same as the test papers before the experimental class of the respective groups. In the same testing stage, Group A and Group B used the same test papers. Similarly, Group C and Group D used the same other test papers. In order to ensure fair and effective results, a senior physician ensured that the difficulty levels of the Group A and B, as well as the Group C and D, were similar during the same stage of testing. Each test should be completed independently and should take no more than 10 min. The paper consisted of 3 parts, namely bone and joint injury, chronic osteoarthropathy and bone tumor. There were 5 questions in each section, so there were 15 questions in the paper. The total score was 150. All questions were set in accordance with the syllabus to ensure that they were different from those in SPARK case database.

SPSS 25.0 statistical software was used to analyze the data. The data of each group obeyed the normal distribution. The independent sample T-test was used to compare the scores of students in Group A and B in each test, also the number of correct answers in each part of the test before and after the experimental class, to see if there were statistical differences. The results of students in Group C and D were counted in the same way. The measurement data were expressed in (x ± s), and *P* < 0.05 indicated that the difference was statistically significant.

## Results

There were no significant differences in gender and age between Group A and B, Group C and D (*P* > 0.05). See Table 1 for details.The attendance rate and test completion rate of all students were 100%. The two average completion rate of SPARK of students in Group A were 96.4% and 92.8% respectively, and Group C were 97.2% and 93.7%. The completion rate of all students was above 90%, and no one voluntarily quit. Therefore, nobody was excluded from this experiment. There were no statistical differences between the average scores of students in Group A and B, Group C and D after theoretical class(*P* > 0.05). In the tests before, after and one week after the experimental class, the average scores of the experimental group were higher than those of the control group. There was no statistical difference between the scores of Group A and B after the experimental class(*P* > 0.05), and there were statistical differences in other scores(*P* < 0.05). The range of lowest and highest scores obtained by Group A and C were higher than or equal to those in Group B and D respectively. In the tests one month after the experimental class, the scores of Group A and B, Group C and D were close to each other, and the differences were not statistically significant(*P* > 0.05). See Tables 2 and 3 for details.

**Table 1:** Age and Gender of students in 4 groups

Group		Total (*n*)	Gender			Age			
			Male: female	*X* ^*2*^	*P*	Age range	Average age	*t*	*P*
Group A	The experimental group	29	10:19	0.648	0.421	20–23	21.41 ± 0.68	1.660	0.104
Group B	The control group	29	13:16			21–22	21.17 ± 0.38		
Group C	The experimental group	30	14:16	0.907	0.341	21–23	21.53 ± 0.57	0.765	0.447
Group D	The control group	29	10:19			21–23	21.41 ± 0.63		

**Table 2:** Comparison of test scores among four groups of students

	Group A (range, mean ± SD values)	Group B (range, mean ± SD values)	t	*P*	Group C (range, mean ± SD values)	Group D (range, mean ± SD values)	t	*P*
Results after the theoretical class	40–150(100.0 ± 25.4)	60–150(101.0 ± 23.8)	−0.160	0.873	30–150(94.7 ± 23.7)	60–130(92.1 ± 18.6)	0.467	0.642
Results before the experimental class	50–120 (84.1 ± 17.4)	30–100(72.1 ± 21.3)	2.363	0.022	60–130(94.0 ± 17.3)	30–130(72.8 ± 25.5)	3.755	0.000
Results after the experimental class	70–130(107.6 ± 14.3)	60–130(102.1 ± 18.0)	1.292	0.202	60–140(107.3 ± 20.3)	50–130(93.1 ± 20.9)	2.652	0.010
Results one week after the experimental class	50–140 (89.7 ± 24.3)	30–120(66.6 ± 23.2)	3.706	0.000	60–130(100.3 ± 19.7)	30–120(77.2 ± 24.0)	4.039	0.000
Results one month after the experimental class	30–130(86.6 ± 28.8)	40–120(84.5 ± 24.0)	0.297	0.767	60–130(95.7 ± 20.3)	40–130(91.7 ± 23.0)	0.699	0.487

**Table 3:**
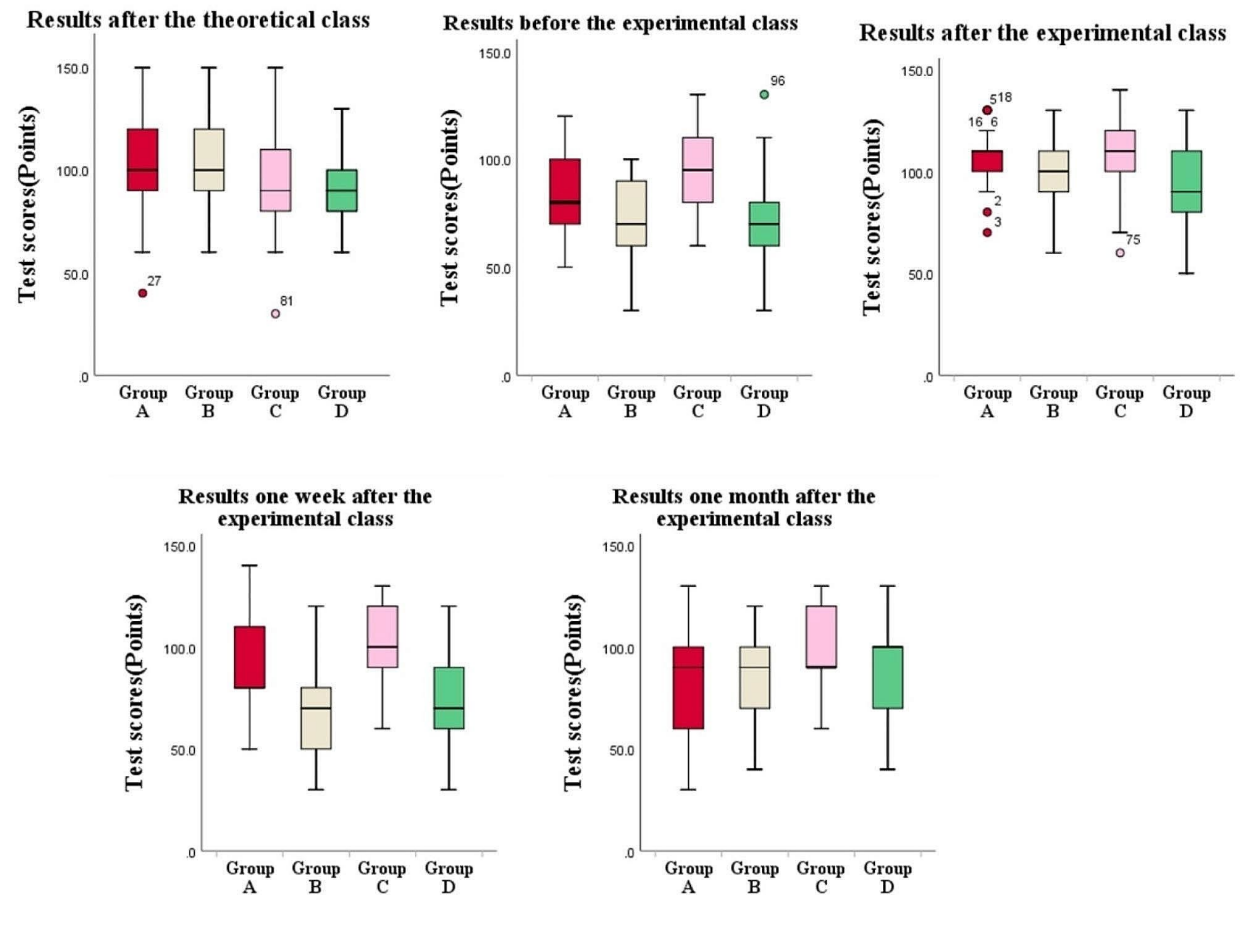
Box plots of the scores of the four groups of students in each test

In the first survey questionnaire, 89.6% of Group A had an average learning time of less than 30 min per day, 96.5% of Group B, 83.3% of Group C, and 86.2% of Group D. The learning time between 30 min and 60 min was 10.3%, 3.4%, 10%, and 6.9%, respectively. No more than 5% of Group C and D studied for more than 60 min. In the second survey questionnaire, 89.6% of Group A had an average learning time of less than 30 min per day, 89.6% of Group B, 83.3% of Group C, and 86.2% of Group D. The learning time between 30 min and 60 min was 6.9%, 10.3%, 10%, and 3.4%, respectively. No more than 10% of Group C and D studied for more than 60 min.

We counted the number of correct answers in each part of the test paper before experimental class, and found that there were no statistical differences between the number of correct answers of students in Group A and B, Group C and D in bone and joint injury and chronic osteoarthropathy(*P* > 0.05), while there was a statistical difference between the number of correct answers in bone tumors(*P* < 0.05). We found that there were many mistakes in the diagnosis and differential diagnosis of bone tumors among the students in 4 groups, especially the confusion between osteosarcoma and suppurative osteomyelitis. The students in Group B also had difficulties in the differentiation of bone cysts and giant cell tumors of bone. Group B and D were deficient in the diagnosis of inconspicuous fracture and joint dislocation, and they also had poor understanding of the imaging manifestations of rheumatoid arthritis and ankylosing spondylitis. Therefore, in the experimental class, the teachers not only completed the content required by the syllabus, but also made additional explanations for the deficiencies of each group of students. After the experimental class, students in all groups improved their mastery of the corresponding sections, and there were no statistical differences in the mastery of each part(*P* > 0.05), as shown in Table 4. In addition, compared with the control group, students in the experimental group showed more confidence and higher participation and activity in class.

**Table 4:** The number of correct answers in each part of the test paper before and after the experimental class

		Group A	Group B	t	*P*	Group C	Group D	t	*P*
before the experimental class	bone and joint injury	3.0 ± 0.8	2.9 ± 1.2	0.654	0.516	3.2 ± 1.1	2.7 ± 1.3	1.400	0.167
	chronic osteoarthropathy	3.0 ± 0.8	2.8 ± 1.1	0.925	0.359	3.1 ± 1.0	2.6 ± 1.1	1.896	0.063
	bone tumors	2.5 ± 0.9	1.7 ± 0.8	3.510	0.001	3.0 ± 1.3	2.1 ± 1.2	2.585	0.012
after the experimental class	bone and joint injury	3.7 ± 0.8	3.6 ± 1.2	0.512	0.610	3.8 ± 0.8	3.2 ± 1.2	2.111	0.040
	chronic osteoarthropathy	3.4 ± 0.8	3.3 ± 1.2	0.130	0.897	3.3 ± 0.9	3.1 ± 1.2	0.579	0.565
	bone tumors	3.5 ± 1.2	3.3 ± 1.0	0.853	0.397	3.6 ± 1.1	3.1 ± 1.0	1.848	0.070

## Discussion

In this study, teachers assigned different learning tasks to different groups of students, took certain measures to supervise, and conducted tests at different stages and analyzed their scores, aiming to compare the application effect of traditional teaching model and process-based teaching based on SPARK case database in the practice teaching of radiology in the musculoskeletal system for undergraduate medical students.

The current changing global healthcare environment poses great challenges for medical education [10–12]. To meet the development and needs of the new era, such as case-based learning(CBL), problem-based learning(PBL), flipped classroom(FC) and other new and effective teaching methods have been incorporated into the university curriculum [13–18]. CBL requires students to think and communicate around clinical cases, PBL requires them to consult materials to solve problems, and FC readjusts their learning time in and out of class, which increases students’ autonomy in learning. Admittedly, these methods have improved the learning effect of students to a certain extent, but there are still some limitations. For undergraduates, radiology is a new subject, CBL and PBL have great difficulties. The over-detailed methods are more suitable for students with a certain knowledge reserve of imaging. FC gives students more freedom, but the lack of guidance and supervision from teachers will reduce the enthusiasm of some students. In addition, the combination of various new teaching models has also been gradually applied. A study believed that the combination of PBL and team-based learning(TBL) teaching methods could achieve the purpose of optimizing students’ learning methods [19]. Liu said that the combination of PBL and CBL was more effective for students in the dental field to learn complex surgical skills [20]. Antonis’s research showed that the teaching strategy of TBL combined with FC improved students’ participation in learning and achieved better results [21]. To master the subject of radiology needs a long time of clinical practice. Classroom teaching cannot combine theory with practice at all times. The mixed teaching methods lack the tracking of students’ follow-up learning effect, and cannot become a means of realizing process-based teaching. By optimizing the combination of the above types of new teaching models and combining with our own actual needs, our team innovatively established the SPARK case database for public welfare purposes, providing a digital and intelligent simulation online teaching platform for medical imaging teaching. After installing the mobile application, teachers can operate on their computers or phones to build online teaching resources, and publish online courses, videos, teaching tasks, assignments, exams, voting, and discussions. Students can log in to the client to complete learning and various tasks assigned by the teacher. Teachers and students can also interact and communicate through the platform. Relying on this teaching platform, the mixed online and offline teaching mode can be applied in practice, with the advantage of conducting learning without being limited by location. Its establishment has broken through the bottleneck of uneven distribution of educational resources and is a powerful exploration in the reform of medical teaching model in our department.

In the test after theoretical class, the average scores of students in Group A and B were close to each other, as were those in Group C and D, which proved that students in the experimental group and the control group had roughly similar listening effects in theoretical class, and their imaging fundamentals were basically at the same level. In the test before experimental class, the results of the experimental group were better than those of the control group, which indicated that different teaching models caused a preliminary gap in students’ scores. Students in the experimental group could use SPARK case database at any time on their mobile terminals, so that they could make full use of fragmented time and arrange learning process in a more personalized and free way, then learning was no longer restricted by space and time. In addition, the five links of SPARK were the embodiment of the “vertical process” of learning: S emphasized the basic theoretical system of musculoskeletal system. P further deepened the memory of basic knowledge by students searching for answers to given questions. A and R links gradually applied the theory into practice and simulated the workflow of radiological diagnosis. K integrated radiology knowledge with various clinical disciplines to help students analyze cases from a macro perspective. The completion of the five links of learning would help students’ learning ability to get a spiral climb, but also could enrich the students’ learning experience, improve the enthusiasm of learning. It is worth mentioning that in the learning process of the experimental group, the teacher’s supervision played a huge role. In China, students tend to make a better impression on teachers. Therefore, on the premise that the learning process could be observed by teachers in the background, more students voluntarily and timely completed the learning task, although they were completely free to choose whether to do so or not. The above mentioned may be the reason why the experimental group scores higher than the control group.

We noticed that in the test before the experimental class, the difference between the scores of Group A and B was smaller, while the difference between Group C and D was larger. In the subsequent experimental class, in addition to explaining the knowledge points required by the syllabus, the teacher also focused on the diagnosis and differential diagnosis of bone tumors according to the results of the pre-class test, repeatedly emphasizing the differences between osteosarcoma and suppurative osteomyelitis, and the differences between bone cysts and giant cell tumors of bone. Also, the teacher explained how to observe inconspicuous fractures for students of Group B and D, and emphasized the imaging manifestations of rheumatoid arthritis and ankylosing spondylitis. In the test after the experimental class, the average score of Group C was still higher than that of D and there was statistical difference, but there was no statistical difference between Group A and B. The author believes that the reasons for the above phenomenon are as follows: between the theoretical class and the experimental class, Group A and B only had 3 days to learn. The experimental group using the new teaching model had a slightly better learning effect than the control group using the traditional method. In the subsequent experimental class, the teacher made up the gaps, and the small advantages formed by the experimental group in the 3 days were erased, so the results of the test after experimental class were similar. Different from the former, after 10 days of learning in different models of Group C and D, the experimental group got better results, and the difference between the two groups was large. Although the teacher’s explanation in the experimental class reduced the achievement gap between the two groups in the after-class test, the experimental group gained a big advantage after a long period of learning in the new teaching model, which could not be eliminated by the 4 h experimental class. Therefore, the score of Group C was still higher than that of D in the test after the experimental class. From the results of two survey questionnaires, it could be seen that the majority of students spent less than 30 min or between 30 and 60 min studying medical imaging per day, with little difference in distribution. Perhaps in the following experiments, the experimenter should refine the duration of the survey questionnaire to observe the impact of learning time on the results. According to Table 4, we can find that there were no statistical differences in the number of correct answers of students in the test before the experimental class from Group A and B, Group C and D in bone and joint injury and chronic osteoarthropathy. The reason may be that the contents of these two parts were relatively simple and easy to understand, and the learning effects of different teaching models were not different. However, there was a statistical difference in the number of correct answers in the part of bone tumor. Because bone tumors were difficult, the students in the control group had a certain difficulty in integrating complex knowledge through books and courseware, so their mastery was not as good as that of the students in the experimental group. Interestingly, in the test after the experimental class, students in Group C and D only had difference in the part of bone and joint injury, while there were no differences in the correct answers of other parts, and the average scores were different. Perhaps the reason was that although Group C gained an advantage that could not be remedied, the teacher still played a certain role in the learning process, leading Group C to have only small advantage over D in each part. Finally, after another week of learning, Group A and C obviously got better results than Group B and D in the test one week after the experimental class. Finally, it should be emphasized that clinical teaching was not a pure scientific research experiment, the teaching quality should be the core of everything. Therefore, based on the principle that education should be fair and high-quality, the experimenters provided all the musculoskeletal system question set for each group of students, so that they could have equal access to learning opportunities. After a certain period of study, the results of the final test which was conducted one month after the experimental class showed that the scores of students in Group A and B, Group C and D were basically close. In this experiment, SPARK case database ran through every stage of learning, including after-class review of theory (preview before experimental class), after-class review of experimental class and review after a certain period of time of experimental class, which reflected the “horizontal process” of learning. To sum up, learning based on SPARK case database was a comprehensive procedural learning both vertically and horizontally. This method ensured that there were rules to follow in every link, made teaching management simple and transparent, which proved that the application of SPARK case database could become an effective means to realize process teaching.

There were still some deficiencies in this experiment: ① This experiment was a single center study, and the research content was limited to the imaging of musculoskeletal system; ② The study time in the questionnaire survey should be set more reasonably, resulting in a concentrated distribution of students’ choices, and it was impossible to accurately compare the study time of the two groups of students; ③ SPARK learning task may aggravate students’ load to some extent, but some literature suggested that compared with students in the traditional model, students in the new teaching model spent more time on homework per week, but less time on exam preparation [22–24]. ④Under the arrangement of the school, the interval between the theoretical and experimental classes was different, so the random experiment couldn’t be carried out in this experiment. In subsequent research, we will conduct random experiments on students who attend classes at the same time. Therefore, more perfect experimental scheme should be designed for further research in the future.

In conclusion, the process-based teaching based on SPARK case database had achieved good application effect in the practice teaching of radiology in the musculoskeletal system for undergraduate medical students. It is helpful for students to transform theoretical knowledge into practical application ability quickly and effectively, and helps to cultivate their self-learning ability and disease diagnosis ability.The successful application of this model in musculoskeletal system provides a good reference and demonstration, and provides a new method for imaging teaching and medical teaching in other systems. In the future, the process-based teaching based on SPARK case database may even become a lifelong learning model for medical students.

## Data Availability

The datasets used and analysed during the current study are available from the corresponding author on reasonable request.

## Abbreviations

S: Sub-speciality
P: Problem-based learning
A: Assessment
R: Report
K: Reading skill
Ta-t: Test after the theoretical class
Tb-e: Test before the experimental class
Ta-e: Test after the experimental class
Tw-e: Test one week after the experimental class
Tm-e: Test one month after the experimental class
CBL: Case-based learning
PBL: Problem-based learning
FC: Flipped classroom
TBL: Team-based learning
